# The Link between Subjective Religiosity, Social Support, and Mental Health among Young Students in Eastern Europe during the COVID-19 Pandemic: A Cross-Sectional Study of Poland and Ukraine

**DOI:** 10.3390/ijerph19116446

**Published:** 2022-05-26

**Authors:** Piotr Długosz, Damian Liszka, Luydmila Yuzva

**Affiliations:** 1 Institute of Philosophy and Sociology, Pedagogical University of Krakow, 30-084 Krakow, Poland; damian.liszka@up.krakow.pl; 2 Department of Sociology, Taras Shevchenko National University of Kyiv, 01033 Kyiv, Ukraine; luydmilay@ukr.net

**Keywords:** COVID-19 pandemic, subjective religiosity, social support, mental health, philosophy of religion, students, Poland, Ukraine

## Abstract

Religiousness has a positive effect on the mental health of an individual and social groups in many difficult situations. In the conducted research, we wanted to check, inter alia, whether religiosity and social support are positively related to the mental health of students during the COVID-19 pandemic in Poland and Ukraine. The research was conducted at a time (August 2021) when the very contagious Delta variant was spreading over Europe, and numerous pandemic-related personal restrictions and obligations (such as using facemasks in selected places, social distancing, and obligatory self-isolation of the ill or those who had contact with the pathogen) were in force in both countries. For this purpose, a representative survey was carried out using the CAPI technique on a sample of 1000 students in Poland (50% boys and 50% girls in the age range 10–19) and 1022 in Ukraine (51% boys and 49% girls in the age range 10–18). The results of the research shows that depression measured by the PHQ-9 scale was experienced by 20% of students in Poland, and 13% in Ukrainian. Anxiety, measured with the GAD-7 scale, was experienced by 9% of the Polish and 6% of the Ukrainian students. The performed regression analysis showed that religiosity had no effect on the mental health of students. The main risk factor for mental disorders was the lack of social support.

## 1. Introduction

Religiosity is a concept that is difficult to define, since, in addition to purely terminological issues, it crosses several academic disciplines, such as theology, philosophy, psychology, and sociology. Each of those disciplines considers religiosity from a different point of view [1,2,3]. Theologists analyze it from the viewpoint of faith, psychologists as an aspect of personality, and sociologists view religiosity as church membership and attendance, doctrinal knowledge, and living the faith [1,2,4,5].

On the other hand, the philosophy of religion focuses on religiosity as a religious experience and analyzes aspects of it, such as the following: the perception of God [6], the way of life of a religious person, the way of looking at the world as a whole that prepares an individual foundation for religion, and other cultural pursuits such as science or art [7], or the reference of general philosophical theory to religion using different approaches and methods, such as a phenomenological-existential one [8].

Many studies have been performed examining the relations of the different dimensions of religiosity, mental health, and life satisfaction. Smith et al. [9], in a meta-analysis of 147 studies, confirmed that greater religiousness is associated with fewer depression symptoms. Despite the overall findings, the results were dependent on the type of religiousness, with extrinsic religiousness and negative religious coping associated with higher levels of depressive symptoms.

Findings of some research seem to conclude that aspects of religiosity, such as church attendance or personal prayer, are important in relation to anxiety-buffering, e.g., [10,11,12], also among students [13,14]. It is also noticeable that, during the COVID-19 pandemic, mental health is partially protected by religiosity in the adult population. This is confirmed by research carried out in Brazil where religiosity was found to have an important role in the relief of suffering, minimizing the consequences of social isolation, and resulting in better mental health outcomes [15]. Another study found significantly higher levels of positive religious coping among the Muslims than the Christian residents of the United Arab Emirates during the COVID-19 pandemic [16]. A study on 419 adult American Orthodox Jews found that positive religious coping was associated with lower stress levels [17]. A number of other studies confirm that religiosity could be a tool used to deal with the new pandemic reality [18,19,20].

A meta-analysis of 850 studies by Koenig and Larson [21] found evidence supporting a positive relationship between religiosity, mental health, social support, and life satisfaction. Nonetheless, the authors also presented studies that concluded an association between religion and worse mental health. There were also studies that concluded that religion positively correlated with the everyday psychological adjustment [21,22].

In the case of adolescents and young adults, research on the link between mental health and religiosity appears much less frequently. This was most likely because questions of religion can be perceived as culturally sensitive in a number of countries. A review of 20 articles between 1998 and 2004 by Wong et al. [23] found that most studies (90%) showed that higher levels of religiosity in adolescents (in three aspects: “institutional”, “ideological”, and “personal devotion”) were associated with better mental health. Another study shows that there is a small positive correlation between happiness and religiosity [24]. It was also noted in the research of students that intrinsic religiosity was negatively related to depressive symptoms [25]. Similarly, in the case of Muslim adolescents, a positive relation between well-being and positive religious coping methods was noticed [26].

Not many researchers, so far, have focused strictly on subjective religiosity. This dimension usually refers to perceptions and attitudes towards religion and is measured by questions of the role of religious beliefs in day-to-day activities, perceived importance of religion, or subjective perception of being religious [27,28]. Some studies on the subjective perception of one’s own religiosity and the connection to other dimensions of religiosity have been conducted [27,29,30]. Many of the studies have found that intrinsic religiousness, more than extrinsic orientation, is associated with psychosocial adjustment [13,24,30]. Batson developed a three-factor model of religious orientations “as a means (extrinsic), end (intrinsic), and quest” [31] (p. 38). The third dimension, which defines religion as an expression of an open-minded search for truth [32], is less frequently used in research examining the relationship between religious orientation and psychological well-being [14,33], probably due to its recent formation [34]. Some studies report no significant relationship between quest religious orientation and psychological well-being measures [14,35], while others found a positive correlation between quest orientation and depression [36] or anxiety [37]. Lavrič and Flere [13] observed a significant negative correlation of quest orientation with at least one dimension of psychological well-being in samples from five different cultural/religious environments: Slovenia, Bosnia and Herzegovina, Serbia, the United States of America, and Japan.

Although a number of empirical investigations have found links between aspects of religiousness, social support, and mental health, most of them focus on samples from the USA or other English-speaking countries [13,38]. Snoep [38] concluded that the correct question in further research should not be if religiousness boosts happiness, but in what conditions and for what kind of people does it boost happiness. The importance of determining which aspects of religiosity correlate with life satisfaction, and if the correlations are different for people of different religions, is also pointed out by Cohen [39]. Results of the meta-analysis of 90,000 individuals in 26 European nations using the European Social Survey show that people are more satisfied in more religious regions while atheistic regions make religious and atheist people less satisfied [40]. Another study by Okulicz-Kozaryn [41], points out that religiosity makes people happier; however, it seems to only have that effect in religious nations [41]. Poland and Ukraine are examples of religious societies. According to the World Values Survey, 83% of the population in Poland is considered religious, and 72% in Ukraine. A total of 85% of people in Poland, and 70% in Ukraine, participate in religious practices at least once a year [42].

The aim of this research is to check whether subjective religiosity and social support have an impact on the mental health of adolescents among religious societies in Central and Eastern Europe.

The adolescent age group was selected for this study, not without a reason. Many studies show that this age group suffered the most from a deterioration of mental health during the COVID-19 pandemic [43,44]. The hypothesis adopted for testing indicates that, with an increase in social support and religiosity, mental disorders among students decrease.

## 2. Materials and Methods

### 2.1. Participants

A random sample selection was used to conduct the survey in Poland and Ukraine. A random-stratified method was used, taking into account such selection criteria as household income, place of residence, age, and type of school. The survey research was carried out by means of computer-assisted personal interviewing (CAPI). Ultimately, 1000 interviews were correctly conducted in Poland and 1022 in Ukraine. The research was carried out in both countries during the summer break in August 2021. Although in May and June 2021 some personal restrictions were lifted; at the time of the survey, the very contagious Delta variant was spreading over Europe and numerous pandemic-related restrictions and obligations were in force in both countries. In Poland and Ukraine, social distancing, obligatory self-isolation of the ill or those who had contact with the pathogen, and the use of facemasks in most public places were required. Moreover, new restrictions for people entering the country were introduced in Ukraine on 21 July 2021 (rules for self-isolation, excluding some categories of travelers). Similar restrictions were introduced even earlier in Poland to combat the spread of the Delta variant. Research in Poland was carried out by Research Collective, and in Ukraine, it was carried out by the Kiev International Institute of Sociology. The participants did not get an online link to the questionnaire, however, they were interviewed in face-to-face interviews, taking into account generally recognized international ethical standards for human research, such as the European textbook on ethics in research (EC 2010), recommendations by the Helsinki Human Rights Organization and were in accordance with the laws of the location. Ethics committee approval was obtained in both countries.

We calculated the means, SD and % of the variables. We used Pearson’s correlation, one- and two-sample *t*-tests, and multiple regression to test the hypotheses. IBM SPSS was adopted for analyzing data.

### 2.2. Demographic Characteristics

The research took into account demographic and social variables, of which a detailed list is presented in Table 1. The obtained data shows that gender was equally distributed in both countries’ samples. The average age of the respondents indicates that the students in the Ukrainian sample are slightly younger. This is due to a different education system in Ukraine in which education ends one year earlier than in Poland. The exact distribution of the age groups was as follows in Poland (10 years—*n* = 5; 11 years—*n* = 104; 12 years—*n* = 130; 13 years—*n* = 115; 14 years—*n* = 126; 15 years—117; 16 years—*n* = 137; 17 years—*n* = 137; 18 years—*n* = 89; 19 years—*n* = 40). In Ukraine, the exact age distribution was as follows (10 years—*n* = 150; 11 years—*n* = 118; 12 years—*n* = 137; 13 years—*n* = 103; 14-years—*n* = 97; 15 years—*n* = 185; 16 years—*n* = 130; 17 years—*n* = 75; 18 years—*n* = 26). Most of the respondents assess their material conditions as average. The comparison of the descriptions of both samples shows that Ukrainian students have a lower assessment of the material conditions than their Polish peers. In both countries, the majority of students live in cities, which is in line with the structure of the general population. An average social status is dominant among the surveyed students, which was measured by the parents’ education, their professional status, and financial situation. The number of books, which were used as an indicator of cultural capital, has a similar distribution in both groups. Material status, measured by the possession of various household items, such as a car, dishwasher, permanent Internet access, etc., is higher among Polish students. Overall, collected characteristics indicate that the adolescents subjected to the research have similar socio-demographic parameters. The only visible and significant difference is that Polish students have better material conditions than their Ukrainian peers.

### 2.3. Subjective Religiosity

Religiosity was measured on an ordinal scale with a subjective declaration of the level of religiosity from non-believer = 1, undecided = 2, believer = 3 and deeply religious = 4. A total of 2% of respondents in Poland and 4% in Ukraine were considered to be deeply religious, 67% in Poland and 74% in Ukraine were believers, 22% in Poland and 13% in Ukraine were undecided, and 10% in Poland and 8% in Ukraine were non-believers.

### 2.4. Short Scale of Youth’s Social Support Assessment

The Short Scale of Youth’s Social Support Assessment (SSYSSA 18) was prepared by Skowroński and Pabich [45] and consisted of 18 items. The scale has three subscales of student, parental and teacher support. The responses on the scale range from definitely no (=1) to definitely yes (=5). The higher the respondent’s score, the more support he/she has. In earlier studies, Cronbach’s alpha reliability was 0.85 [45]. In the present study, Cronbach’s alpha in the Polish sample was 0.90, in the Ukrainian sample, it was 0.91. Thus, excellent psychometric properties are present in both communities.

### 2.5. Trust

Trust in this research is treated as one of the indicators of social capital [46,47]. The scale of trust consists of nine items, among which there is trust in teachers, parents, neighbors, and politicians. Confidence was measured on a scale between definitely do not trust (=1) to definitely do trust (=5). The higher the final score, the higher the level of trust the respondent has. In the conducted research, Cronbach’s alpha in the Polish sample was 0.76, and in the Ukrainian sample, it was 0.82. Trust levels differ in the adult populations of both countries. This may point to either the specificity of the sample in question or some indicators of social changes.

### 2.6. PHQ-9

Patient Health Questionnaire-9 is used worldwide for depression screening. It has proven and good psychometric properties [48]. The validation of the Polish language version was conducted by [49,50]. Participants assess how frequently they were bothered by each symptom over the past 2 weeks, on a 4-point Likert-style rating scale ranging from 0 to 3 (0 = not at all, 1 = several days, 2 = more than half of the days, 3 = nearly every day).

In the present tests, Cronbach’s alpha in the Polish sample was 0.89, and in the Ukrainian sample, it was 0.91. We found that the result placing the respondent in the norm was achieved by 79% of the respondents in Poland and 82% in Ukraine. A total of 12% of the respondents in Poland had a slight depression, and 11% in Ukraine. A moderate state of depression was observed among 6% of Polish surveyed students, and among 4% of Ukrainian students. Moderately severe depression was manifested among 2% of the Polish surveyed community, and 2% of the Ukrainian community. Severe depression was reported by 0% in Poland, and 1% in Ukraine.

### 2.7. Generalized Anxiety Disorder

GAD-7 is a 7-item screening tool for generalized anxiety disorder. Recipients assess how frequently they have been bothered by each of the seven symptoms over the past 2 weeks according to a 4-point rating scale ranging from 0 to 3. (0 = not at all, 1 = several days, 2 = more than half of the days, 3 = nearly every day). In these tests, Cronbach’s alpha in the Polish sample was 0.90, and in the Ukrainian sample, it was 0.85. The result of 5, 10, and 15 points indicate the presence of mild, moderate, and severe anxiety, respectively. Obtaining at least 10 points indicates a high probability of the occurrence of a generalized anxiety disorder [51]. This result, indicating clinical anxiety disorders, was observed among 9% of the Polish students, and among 6% of the Ukrainian students.

### 2.8. Satisfaction with Life Scale

The Satisfaction with Life Scale (SWLS) was proposed by Diener and colleagues to measure life satisfaction [52]. The Polish adaptation was carried out by Jankowski [53]. It consists of five statements with which respondents may or may not agree. In this study, the scale was modified and a 5-point measurement was used instead of the 7-point measurement (1 = strongly disagree, 2 = rather disagree, 3 = neither agree nor agree, 4 = rather agree, 5 = strongly agree). The higher the overall score, the higher the life satisfaction. In these tests, the Cronbach’s alpha in the Polish sample was 0.83, and in the Ukrainian sample, it was 0.80.

## 3. Results

The results of the analysis presented in Table 2 show that there are statistically significant differences with regard to religiosity. Young Ukrainians are more religious than young Poles. This is confirmed in the World Values Survey [42]. The obtained results show that the average value for religious practices was 3.37 in Poland and 4.54 in Ukraine. The result was statistically significant and a strong effect size of Cohen’s d = 0.56 was observed.

In the case of social support and its perceived dimensions, statistically significant differences were observed. In general, social support turns out to be higher among Polish than Ukrainian students. Ukrainian respondents, however, had more support from their parents. In Poland, students had greater support from their peers and teachers. The results of the *t*-test comparisons show that the level of mental health and confidence were the same in both trials.

Subsequent analyses show that, in both countries, there are related variables taken into account. Table 3 presents the results of the analysis of the data from the Polish sample. The correlation analysis shows that as religiosity increases, the scores on the anxiety and depression scale decrease and the scores on the well-being scale increase.

Another factor, even more closely related to mental health indicators, is social support. As social support increases, anxiety, and depression decrease and life satisfaction increases. Parental support is the most closely related to the mental condition of students.

In addition to the key factors listed above, demographic characteristics also had an impact on the mental condition of adolescents. Depression was negatively correlated with the assessment of the financial situation and social status, and positively with the number of books. Anxiety was also negatively correlated with the assessment of the financial situation and social status. Life satisfaction was negatively correlated with age and positively correlated with the assessment of the financial situation and social status. Girls were characterized by higher well-being. It is also worth adding that trust was negatively correlated with depression and anxiety.

The results of the correlation analysis indicate religiosity as being an important protective factor. Students from higher-status families who have trust capital also have better mental conditions. The gender and age of the students were less related to mental health.

The data for Ukraine, presented in Table 4, shows that religiosity is negatively correlated with depression and anxiety. Social support is another factor that has a positive effect on mental health. Student support was the most closely related to depression and anxiety, and parental support was associated with well-being. As in the case of Polish students, demographic characteristics also turned out to be statistically significant. Depression was positively correlated with age and being female, and negatively with the assessment of financial conditions and social status. It is worth mentioning that depression is often connected to the female gender, as men are often misdiagnosed or suffer from other mental disabilities, such as addiction [54]. The same is true of the anxiety correlation. Anxiety is positively correlated with age, female gender, and wealth, and negatively with the assessment of financial conditions and social status. Well-being was positively correlated with wealth and social status. Trust was negatively correlated with depression and anxiety and positively correlated with well-being.

The research results show that social support and trust are the main protective factors for Ukrainian youth. Religion is of lesser importance in terms of providing protection for mental health. Students with a higher social status and better assessment of their financial situation cope better in a pandemic.

Comparing the results of the correlation analysis between the two groups, it should be stated that social support, religiosity, and social status are risk factors for mental disorders in both countries. There are also important differences. Religiousness does not affect the social well-being in Ukraine. In Poland, girls more often have a better well-being than boys. In Ukraine, the female gender is positively correlated with depression and anxiety. Additionally, in Ukraine, there was a link between the wealth of the respondents, anxiety, and well-being. On the one hand, the rich can afford to have their needs met. On the other hand, in a pandemic, wealthier people may fear that the pandemic may impoverish them. In Poland, mental disorders appeared more often among students living in the city. Moreover, in Poland, the relationship between cultural capital, measured by the number of books, trust, and mental disorders, has emerged. While in Ukraine, material factors have a greater influence on the mental condition, in Poland, the influence of cultural factors, i.e., cultural and social capital, is more often observed.

A regression analysis was performed (Table 5) in order to accurately determine the predictors of mental health among students in Poland and Ukraine. In the case of depression, measured with the PHQ9 scale, independent variables explain 22% of the variability in Poland and 13% in Ukraine. In Poland, the level of depression increases with the deterioration of the assessment of the financial situation, the decline in trust level, and declining student and parental support. Depression also increases with the number of books possessed at home. In Ukraine, depression increases for the female gender. Schoolgirls are more depressed. As student and parental support decline, depression increases.

Similar mechanisms were observed in the case of anxiety measured by the GAD7 scale. The percentage of variance explained by independent variables was 22% in Poland and 11% in Ukraine. Among Polish students, higher anxiety was observed among those with a worse assessment of their financial situation, those who had less student and parental support, and those who had a larger collection of books. The low trust level was also correlated with higher anxiety. In Ukraine, as in the case of depression predictors, a higher level of anxiety was observed in girls and students without student and parental support.

The regression models obtained better parameters in the analysis of the predictors of well-being. The level of explained variance by independent variables was 40% in Poland and 29% in Ukraine. Higher psychological well-being in the Polish sample was observed among students with a better assessment of their financial situation and who had student, teacher, and parental support. In Ukraine, well-being was higher among younger students, among respondents with a higher social status, and among the more affluent. Well-being also grew with higher student, teacher, and parental support.

The results of the regression analysis indicate that parental and student support is the main factor positively effecting the mental condition of students in both countries. Teacher support is only a predictor of student well-being.

The regression analysis results confirm the previously observed differences between the two countries. In Poland, a statistically significant predictor of mental health was the assessment of material conditions. The worse this assessment, the worse the mental condition. The influence of cultural capital on depression and anxiety, measured by the number of books, was revealed again. Trust also had an impact on depression and anxiety.

In Ukraine, the female gender is one of the risk factors for mental disorders. The girls had higher scores on the depression and anxiety scales. A difference also emerged in the case of well-being. Contrary to the Polish sample, well-being was determined by age, social status, and the level of wealth. This could mean that the soul of students was grounded, as written by Czapiński [55], meaning that in post-communist countries, psychological well-being depends more on the material situation, which is also visible among students from poorer countries, such as Ukraine.

## 4. Conclusions

The aim of this study was to find risk factors for mental health disorders among youth in Central and Eastern Europe during the pandemic. Based on many studies conducted around the world, we know that long periods of staying in front of a computer screen, isolation from peers, and restrictions on freedom and privacy contributed to an increase in the level of mental disorders among students [56,57]. In our research, 21% of the respondents in Poland, and 18% in Ukraine, self-declared symptoms of depression. 9% of the respondents in Poland, and 6% in Ukraine, self-declared anxiety symptoms. These results are significantly lower than those of the Chinese adolescent population during the COVID-19 pandemic, where depression was observed in 44% and anxiety in 37% [58]. The results of the meta-analysis of studies conducted around the world among adolescents during the COVID-19 pandemic indicate that depression was present among 21.2–29.7% (95% CI) and anxiety among 17.2–24.4% (95% CI) [59]. Comparing our results to those presented above, it should be noted that the symptoms of depression among young people from Central and Eastern Europe are similar. The level of anxiety is much lower than that observed in the meta-analysis. Perhaps the lower level of anxiety among adolescents from Poland and Ukraine was due to the timing of the study. The study was completed during the summer break when students are usually relaxed and do not think about problems associated with their student life. The results could differ if the research was carried out during the school year and during distance education. It is worth adding that the researchers observed that higher levels of mental disorders in children and adolescents appeared at the beginning of the pandemic [60].

The results of the regression analysis allowed for the identification of the main risk factors of mental health disorders among adolescents, which include social support, specifically student support and parental support. The positive impact of social support on adolescents has been confirmed in many studies [61,62]. Previous studies also show that parental support was a factor in protecting the mental health of students during the pandemic [61,63]. Social support is effective in protecting mental health as family members or friends reduce anxiety and depression levels in adolescents by using empathy [64]. Social support also improves the individual’s sense of self-efficacy and leads to greater understanding, courage, and self-fulfillment, which can help the individual maintain relatively stable emotions even when difficult situations arise [65]. It is worth adding, however, that our research showed no influence of teacher support on depression and anxiety, however, it was evident in well-being. Several studies have also found a positive effect of teacher support on the mental health of adolescents [66,67]. It is possible that the lack of a relationship between teacher support and anxiety and depression was due to the fact that the students were away from school for over a year while this study was being conducted, hence there was a natural loosening of ties with teachers. Teacher support is an important factor in protecting the mental health of adolescents when parental and peer support is lacking [67]. In the case of Poland and Ukraine, ties with parents and peers are strong [68], hence, teacher support was not of great importance in protecting students from negative emotions.

In the case of Polish students, the financial situation had a strong impact on mental health. A worse assessment of the household’s financial situation increased the risk of mental disorders, which is consistent with other studies [69]. There was also a positive influence on cultural capital measured by the number of books. This can be explained by the fact that students with higher cultural capital have greater aspirations, and for them, remote education. For those students, the pandemic may be an obstacle to the implementation of these plans and may constitute a macrostressor. Students with low aspirations have a different attitude and are happy to have a break from studying [70].

In the case of the Ukrainian sample, a slight influence of age on well-being was observed. Older students had lower well-being than younger students, which can be explained by the fact that older students experience hormonal changes in adolescence and they may experience greater pressure pertaining to their school achievement [71]. The female gender was a risk factor for depression and anxiety among Ukrainian youth, which is consistent with many studies conducted during the pandemic [72]. This phenomenon is associated with the occurrence of greater internalization problems for girls [73]. Our research also shows that the level of trust is negatively associated with depression and anxiety among Polish students. On the one hand, this may prove that social capital, one factor of which is trust, protects adolescents from mental health problems [46,47]. On the other hand, reduced trust is an indicator of socio-cultural trauma [74]. The pandemic is undoubtedly a cultural trauma that causes a lot of negative phenomena [75].

The question of religiosity still remains to be discussed. It was assumed that religiosity, measured by a subjective sense of faith, would be positively related to the mental health of adolescents. This is partly what happened when we take into account the results of the correlation analysis among Polish and Ukrainian students. With an increase in subjective religiosity, the indicators of depression and anxiety decreased. These results were consistent with the results of a meta-analysis, which showed that 90% of the analyzed articles indicated a positive relationship between religiosity and mental health [23]. Confirmation of this impact can be found in the studies of high school students carried out at the beginning of the pandemic in April 2020 in Poland and Ukraine. In Poland, life satisfaction was declared by 81% of deep believers, 72% of believers, 59% of the undecided and 55% of non-believers (*n* = 1492, r = 0.20, *p* = 0.000). In Ukraine, similar results were obtained and life satisfaction was declared by 90% of deep believers, 83% of believers, 74% of the undecided, 64% of non-believers (*n* = 2046, r = 0.19, *p* = 0.000) [76]. Regression analysis in this study did not show the impact of the subjective sense of religiosity on mental health of adolescents. This phenomenon could have been influenced by the fact that in our research, half of the research sample were younger respondents, i.e., primary school students. According to researchers, the relationship between religiosity and mental health is stronger among older adolescents, as attitudes towards religion more often reflect the individual choices of adolescents rather than the set of imposed values and parents’ expectations [77]. Perhaps this resulted in the lack of statistical significance of subjective religiosity with the control of the other variables. Perhaps there are other reasons that should be looked into in future research.

## 5. Limitations

Some limitations of the study need to be highlighted. First, our focus is on only two European countries (Poland and Ukraine) where the majority of the population adheres to Christianity. Whether the results can be generalized to other European countries and to other religions remains to be established. Methodologically, we used only a single indicator as a measure of subjective religiosity. Focusing on only one aspect does not reflect the multiple dimensions of religious experience. Another limitation is that our study focuses on a specific time: August 2021. At that time, the adolescent respondents (students) were exhausted after the prolonged periods of remote learning and the research was done during the summer vacation in both countries. It is to be confirmed if the links between religiosity, social support, and mental health are present in further periods of the pandemic, for example, after students return to school learning. This question remains open to further research. Moreover, the research in both countries was subcontracted to research companies. The authors did not have full control over the quality checking during the realization and data collection in the study.

## Figures and Tables

**Table 1:** Socio-demographic characteristics of participants.

		Poland, *n* = 1000	Ukraine, *n* = 1022
Age	Mean (SD) Min-Max	M = 14.65, SD = 2.35 10–19	M = 13.46, SD = 2.34 10–18
Gender% (*n*)	Female	50 (502)	49 (496)
	Male	50 (498)	51 (526)
Financial standing% (*n*)	Bad	9 (85)	33 (318)
	Average	61 (617)	57 (586)
	Good	30 (298)	10 (96)
Place of residence% (*n*)	Village	41 (414)	32 (331)
	City	59 (586)	68 (691)
Social status	Low	21 (197)	23 (219)
	Medium	66 (611)	60 (574)
	High	12 (115)	17 (170)
Number of books	0–25	31 (304)	33 (334)
	26–200	62 (607)	61 (623)
	Over 200	6 (59)	6 (65)
Material status	Low	9 (92)	51 (523)
	Medium	59 (592)	46 (467)
	High	32 (315)	3 (32)

**Table 2:** Distribution of the values of the variables and the results of the *t*-test.

		Poland	Ukraine	Differences *p*-Value
Religiosity	Mean (SD)	M = 2.59, SD = 0.68	M = 2.74, SD = 0.66	Cohen’s d = 0.22
	Min-Max	1–4	1–4	0.000
Teacher support	Mean (SD)	M = 19.8, SD = 3.74	M = 18.7, SD = 4.56	Cohen’s d = 0.02
	Min-Max	5–25	5–25	0.000
Parental support	Mean (SD)	M = 22.2, SD = 2.99	M = 22.7, SD = 3.05	Cohen’s d = 0.01
	Min-Max	9–25	5–25	0.000
Student support	Mean (SD)	M = 33.7, SD = 4.68	M = 31.2, SD = 6.28	Cohen’s d = 0.04
	Min-Max	10–40	8–40	0.000
Social support	Mean (SD)	M = 75.8, SD = 9.37	M = 72.7, SD = 11.52	Cohen’s d = 0.02
	Min-Max	34–90	22–90	0.000
Trust	Mean (SD)	M = 33.3, SD = 6.42	M = 33.2, SD = 5.89	Cohen’s d = 0.00
	Min-Max	11–45	12–45	0.715
PHQ-9	Mean (SD)	M = 2.7, SD = 4.03	M = 2.5, SD = 3.97	Cohen’s d = 0.00
	Min-Max	0–26	0–26	0.261
GAD-7	Mean (SD)	M = 2.5, SD = 4.15	M = 2.4, SD = 3.61	Cohen’s d = 0.00
	Min-Max	0–20	0–21	0.563
SWSL	Mean (SD)	M = 18, SD = 3.36	M = 18.1, SD = 3.75	Cohen’s d = 0.00
	Min-Max	5–25	5–25	0.528

**Table 3:** Correlation coefficients among all predictors (Poland).

Variables	1	2	3	4	5	6	7	8	9	10	11	12	13	14	15
1. Religiosity		-													
2. Age	0.21 **														
3. Gender	0.09 **	0.00													
4. Evaluation of financial status	0.08 **	0.00	0.03												
5. Place of residence	0.13 **	0.01	0.03	0.02											
6. Social status	0.04	0.01	0.07 *	0.66 **	0.18 **										
7. Number of books	0.10 **	0.01	0.06 *	0.12 **	0.11 **	0.27 **									
8. Wealth Level	0.01	0.04	0.06	0.36 **	0.15 **	0.44 **	0.24 **								
9. Student support	0.08 **	0.01	0.08 **	0.16 **	0.03	0.16 **	0.01	0.11 **							
10. Teacher support	0.24 **	−0.08	0.12 **	0.16 **	0.05	0.13 **	−0.07 *	0.16 **	0.49 **						
11. Parental support	0.22 **	−0.15	0.07 *	0.24 **	0.02	0.25 **	0.02	0.18 **	0.49 **	0.51 **					
12. Social support	0.21 **	−0.09	0.11 **	0.22 **	0.00	0.21 **	−0.04	0.18 **	0.85 **	0.81 **	0.77 **				
13. Trust	0.24 **	−0.16	0.00	0.01	−0.12	−0.07 *	−0.08 *	−0.09 **	−0.02	0.09 **	0.09 **	0.05			
14. PHQ9	−0.12 **	0.03	0.00	−0.16	0.10 **	−0.06 *	0.20 **	0.05	−0.20 **	−0.17 **	−0.24 **	−0.25 **	−0.21 **		
15. GAD7	−0.11 **	0.00	0.00	−0.19 **	0.17 *	−0.09 **	0.18 **	0.03	−0.24 **	−0.21 **	−0.37 **	−0.32 **	−0.18 **	0.75 **	
16. SWSL	0.14 **	−0.07 **	0.11 **	0.24 **	0.05	0.20 **	−0.03	0.16 **	0.44 **	0.49	0.57 **	0.60 **	0.04	−0.31 **	0.26 **

* *p* ≤ 0.05; ** *p* ≤ 0.01.

**Table 4:** Correlation coefficients among all predictors (Ukrainian).

Variables	1	2	3	4	5	6	7	8	9	10	11	12	13	14	15
1. Religiosity		-													
2.Age	−0.02														
3. Gender	0.02	−0.02													
4.Evaluation of financial status	0.00	0.01	−0.04												
5. Place of residence	0.16 **	−0.00	−0.00	−0.08 **											
6. Social status	−0.01	0.03	−0.01	0.63 **	−0.24 **										
7. Number of books	0.16 **	0.01	0.02	0.11 **	−0.03	0.21 **									
8. Wealth Level	−0.06 *	0.05	−0.03	0.21 **	−0.13 **	0.35 **	0.18 **								
9. Student support	0.08 **	−0.07	0.04	−0.01	−0.01	0.00	0.10 **	0.03							
10. Teacher support	0.01	−0.16	0.07 *	−0.07 *	0.02	−0.04	0.01	−0.05	0.58 **						
11. Parental support	0.07 *	−0.18	0.02	−0.02	0.00	−0.02	0.08 **	0.05	0.45 **	0.43 **					
12. Social support	0.07 *	−0.15 **	0.05	−0.04	0.00	−0.02	0.08 **	0.00	0.89 **	0.83 **	0.68 **				
13. Trust	0.04	−0.17 **	0.02	−0.02	0.05	0.00	0.02	−0.03	0.56 **	0.61 **	0.37 **	0.65 **			
14. PHQ9	−0.07 *	0.08 *	0.13 **	0.09 **	−0.01	0.10 **	0.03	0.05	−0.25 **	−0.16 **	−0.22 **	−0.26 **	−0.15 **		
15. GAD7	−0.07 *	0.09 *	0.13 **	0.08 **	−0.06	0.12 **	0.01	0.07 *	−0.23 **	−0.17 **	−0.20 **	−0.25 **	−0.17 **	0.79 **	
16. SWSL	0.02	0.02	0.00	0.03	−0.04	0.08 **	0.03	0.09 *	0.38	0.37 **	0.45 **	0.47 **	0.32 **	−0.22 **	−0.18 **

* *p* ≤ 0.05; ** *p* ≤ 0.01.

**Table 5:** Results of regression analysis.

Variables	PHQ9				GAD7				SWSL			
	Poland		Ukraine		Poland		Ukraine		Poland		Ukraine	
	β	t	β	t	β	t	β	t	β	t	β	t
Religiosity	0.02	0.69	0.04	1.41	0.01	0.35	0.04	1.41	−0.02	−1.01	0.01	0.56
Age	−0.05	−1.77	0.05	1.48	−0.05	−1.58	0.05	1.48	−0.00	−0.15	−0.07	−2.77 **
Gender	0.00	−0.25	0.15	4.64 **	0.01	0.35	0.15	4.64 **	0.03	1.49	−0.02	−0.80
Evaluation of financial status	−0.18	5.00 **	0.03	0.892	−0.12	−2.88 **	0.03	0.89	0.10	2.97 **	−0.03	−0.085
Place of residence	0.04	0.13	−0.04	−1.33	0.05	1.72	−0.04	−1.33	−0.03	−1.41	−0.01	−0.36
Social status	0.03	0.92	0.06	1.41	−0.00	−0.12	0.06	1.41	−0.02	−0.56	0.09	2.55 **
Number of books	0.21	6.79 **	0.02	0.73	0.17	5.23 **	0.02	0.46	−0.02	−0.91	−0.04	−1.41
Wealth Level	−0.00	−0.12	0.05	1.41	0.01	0.44	0.05	1.60	0.04	1.40	0.08	2.88 **
Student support	−0.15	−4.28 **	−0.18	−4.19 **	−0.11	−2.86 **	−0.18	−4.19 **	0.14	4.53 **	0.16	4.38 **
Teacher support	0.05	1.40	0.02	0.42	0.05	1.43	0.02	0.42	0.20	6.22 **	0.12	3.32 **
Parental support	−0.20	−5.52 **	−0.11	−2.98 **	−0.31	−70.81 **	−0.11	−2.98 **	0.38	11.66 **	0.29	9.35 **
Trust	−0.18	−5.99 **	−0.03	−0.73	−0.13	−4.15 **	−0.03	−0.73	−0.01	−0.46	0.04	1.09
R^2^	0.22 **		0.13 **		0.22 **		0.11 **		0.40 **		0.29 **	

** *p* ≤ 0.01.

## Data Availability

Data available per request. from the corresponding author.

## References

[1] Holdcroft B.B. (2006). What is Religiosity. Cathol. Educ. A J. Inq. Pract..

[2] Cardwell J.D. (1980). The Social Context of Religiosity.

[3] Demerath N.J., Hammond P.E. (1968). Religion in Social Context.

[4] Kasak E. (2011). Some aspects of religiosity in Science. Balt. J. Eur. Stud..

[5] Groome T.H., Corso M.J. (1999). Empowering Catechetical Leaders.

[6] Alston W., Fales E., Peterson M.L., VanArragon R.J. (2020). Does Religious Experience Justify Religious Belief. Contemporary Debates in Philosophy of Religion.

[7] Motak D. (2012). Georg Simmel’s Concept of Religion and Religiosity. Studia Religiol..

[8] Zdybicka Z.J., Bejze B. (1968). Filozoficzna koncepcja religijności człowieka. The philosophical concept of human religiosity. O Bogu i o Człowieku; About God and about Man.

[9] Smith T., Mccullough M., Poll J. (2003). Religiousness and Depression: Evidence for a Main Effect and the Moderating Influence of Stressful Life Events. Psychol. Bull..

[10] Koenig H.G., Westlund M.S., George L.K., Hughes D.C., Blazer D.G., Hybels C. (1993). Abbreviating the Duke Social support index for use in chronically ill elderly individuals. Psychosomatics.

[11] Hertsgaard D., Light H. (1984). Anxiety, depression and hostility in rural women. Psychol. Rep..

[12] Petersen L.R., Roy A. (1985). Religiosity, anxiety, and meaning and purpose: Religion’s consequences for psychological well-being. Rev. Relig. Res..

[13] Lavrič M., Flere S. (2008). The Role of Culture in the Relationship Between Religiosity and Psychological Well-being. J. Relig. Health.

[14] Maltby J., Lewis C.A., Day L. (1999). Religious orientation and subjective well-being: The role of the frequency of personal prayer. Br. J. Health Psychol..

[15] Lucchetti G., Góes L.G., Amaral S.G., Ganadjian G.T., Andrade I., de Araújo Almeida P.O., do Carmo V.M., Manso M.E.G. (2020). Spirituality, religiosity and the mental health consequences of social isolation during COVID-19 pandemic. Int. J. Soc. Psychiatry.

[16] Thomas J., Barbato M. (2020). Positive religious coping and mental health among Christians and Muslims in response to the COVID-19 pandemic. Religions.

[17] Pirutinsky S., Cherniak A.D., Rosmarin D.H. (2020). COVID-19, Mental Health, and Religious Coping Among American Orthodox Jews. J. Relig. Health.

[18] Del Castillo F.A., Biana H.T., Joaquin J.J.B. (2020). ChurchInAction: The role of religious interventions in times of COVID-19. J. Public Health.

[19] Ferrell B.R., Handzo G., Picchi T., Puchalski C., Rosa W.E. (2020). The urgency of spiritual care: COVID-19 and the critical need for whole-person palliation. J. Pain Symptom. Manag..

[20] Weinberger-Litman S.L., Litman L., Rosen Z., Rosmarin D.H., Rosenzweig C. (2020). A look at the first quarantined community in the USA: Response of religious communal organizations and implications for public health during the COVID-19 pandemic. J. Relig. Health.

[21] Koenig H.G., Larson D.B. (2001). Religion and mental health: Evidence for an association. Int. Rev. Psychiatry.

[22] Gartner J., Larson D., Allen G. (1991). Religious Commitment and Mental Health: A Review of the Empirical Literature. J. Psychol. Theol..

[23] Wong Y.J., Rew L., Slaikeu K. (2006). A systematic review of recent research on adolescent religiosity/spirituality and mental health. Issues Ment. Health Nurs..

[24] Francis L.J., Jones S.H., Wilcox C. (2000). Religiosity and happiness: During adolescence, young adulthood, and later life. J. Psychol. Christ..

[25] Walker R., Bishop S. (2006). Examining a Model of the Relation Between Religiosity and Suicidal Ideation in a Sample of African American and White College Students. Suicide Life Threat. Behav..

[26] Aflakseir A., Coleman P.G. (2011). Initial Development of the Iranian Religious Coping Scale. J. Muslim Ment. Health.

[27] Taylor A., MacDonald D.A. (1999). Religion and the five factor model of personality: An exploratory investigation using a Canadian university sample. Pers. Individ. Differ..

[28] Chatters L.M., Levin J.S., Taylor R.J. (1992). Antecedents and dimensions of religious involvement among older black adults. J. Gerontol..

[29] Roccas S., Schwartz S.H. (1997). Church-state relations and the association of religiosity with values: A study of Catholics in six countries. Cross Cult Res.

[30] Aghababaei N., Błachnio A., Arji M., Chiniforoushan M., Mohammadtabar S. (2015). The relations among well-being outcomes, religiosity, and personality. Polish Psychol. Bull..

[31] Batson C.D., Raynor-Prince L. (1983). Religious Orientation and Complexity of Thought about Existential Concerns. J. Sci. Study Relig..

[32] McFarland S.G., Warren J.C. (1992). Religious orientations and selective exposure among fundamentalist Christians. J. Sci. Study Relig..

[33] Wulff D.M. (1997). Psychology of Religion: Classic and Contemporary.

[34] Maltby J., Day L. (2003). Religious orientation, religious coping and appraisals of stress: Assessing primary appraisal factors in the relationship between religiosity and psychological well-being. Pers. Individ. Differ..

[35] Ryan R.M., Rigby S., King K. (1993). Two types of religious internalisation and their relations to religious orientations and mental health. J. Pers. Soc. Psychol..

[36] Genia V.I.E. (1996). Quest, and fundamentalism as predictors of psychological and spiritual well-being. J. Sci. Study Relig..

[37] Kojetin B.A., McIntosh D.N., Bridges R.A., Spilka B. (1987). Quest: Constructive search or religious conflict?. J. Sci. Study Relig..

[38] Snoep L. (2008). Religiousness and happiness in three nations: A research note. J. Happiness Stud..

[39] Cohen A. (2002). The Importance of Spirituality in Well-Being for Jews and Christians. J. Happiness Stud..

[40] Clark A., Lelkes O. Let Us Pray: Religious Interactions in Life Satisfaction. https://halshs.archives-ouvertes.fr/halshs-00566120.

[41] Okulicz-Kozaryn A. (2010). Religiosity and life satisfaction (A multilevel investigation across nations). Ment. Health Relig. Cult..

[42] EVS/WVS (2021). Joint EVS/WVS 2017–2021 Dataset.

[43] Cao W., Fang Z., Hou G., Han M., Xu X., Dong J., Zheng J. (2020). The psychological impact of the COVID-19 epidemic on college students in China. Psychiatry Res..

[44] Orgilés M., Morales A., Delvecchio E., Mazzeschi C., Espada J.P. (2020). Immediate psychological effects of the COVID-19 quarantine in youth from Italy and Spain. Front. Psychol..

[45] Skowroński B., Pabich R. (2015). Krótka skala oceny wsparcia społecznego młodzieży-konstrukcja i właściwości psychometryczne. Profil. Społeczna Resocjal..

[46] Fukuyama F. (1995). Trust: The Social Virtues and the Creation of Prosperity.

[47] Putnam R.D. (2000). Bowling Alone–The Collapse and Revival of American Community.

[48] Kroenke K., Spitzer R.L., Williams J.B.W. (2001). The PHQ-9: Validity of a brief depression severity measure. J. Gen. Intern. Med..

[49] Tomaszewski K., Zarychta M., Bieńkowska A., Chmurowicz E., Nowak W., Skalska A. (2011). Validation of the Patient Health Questionnaire-9 Polish version in the hospitalized elderly population. Psychiatr. Pol..

[50] Kokoszka A., Jastrzębski A., Obrębski M. (2016). Ocena psychometrycznych właściwości polskiej wersji Kwestionariusza Zdrowia Pacjenta-9 dla osób dorosłych. Psychiatria.

[51] Spitzer R.L., Kroenke K., Williams J.B., Löwe B. (2006). A brief measure for assessing generalized anxiety disorder: The GAD-7. Arch. Intern. Med..

[52] Diener E., Emmons R.A., Larsen R.J., Griffin S. (1985). The Satisfaction with Life Scale. J. Pers. Assess..

[53] Jankowski K.S. (2015). Is the shift in chronotype associated with an alteration in well-being?. Biol. Rhythm Res..

[54] Emslie C., Ridge D., Ziebland S., Hunt K. (2006). Men’s accounts of depression: Reconstructing or resisting hegemonic masculinity?. Soc. Sci. Med..

[55] Czapiński J. (1993). Uziemienie polskiej duszy. Grounding the Polish soul. Kult. Społeczeństwo.

[56] Elmer T., Mepham K., Stadtfeld C. (2020). Students under lockdown: Comparisons of students’ social networks and mental health before and during the COVID-19 crisis in Switzerland. PLoS ONE.

[57] Bignardi G., Dalmaijer E.S., Anwyl-Irvine A.L., Smith T.A., Siugzdaite R., Uh S., Astle D.E. (2021). Longitudinal increases in childhood depression symptoms during the COVID-19 lockdown. Arch. Dis. Child..

[58] Zhou S.J., Zhang L.G., Wang L.L., Guo Z.C., Wang J.Q., Chen J.C., Liu M., Chen X., Chen J.X. (2020). Prevalence and socio-demographic correlates of psychological health problems in Chinese adolescents during the outbreak of COVID-19. Eur. Child Adolesc. Psychiatry.

[59] Racine N., McArthur B.A., Cooke J.E., Eirich R., Zhu J., Madigan S. (2021). Global Prevalence of Depressive and Anxiety Symptoms in Children and Adolescents During COVID-19: A Meta-analysis. JAMA Pediatr..

[60] Hawes M.T., Szenczy A.K., Olino T.M., Nelson B.D., Klein D.N. (2021). Trajectories of depression, anxiety and pandemic experiences: A longitudinal study of youth in New York during the spring-summer of 2020. Psychiatry Res..

[61] Tang X., Tang S., Ren Z., Wong D.F.K. (2020). Psychosocial risk factors associated with depressive symptoms among adolescents in secondary schools in mainland China: A systematic review and meta-analysis. J. Affect. Disord..

[62] Meng Q., Shuang-Jiang Z., Zhao-Chang G., Li-Gang Z., Hong-Jie M., Xiao-Min L., Jing-Xu C. (2020). The Effect of Social Support on Mental Health in Chinese Adolescents During the Outbreak of COVID-19. J. Adolesc. Health.

[63] Crockett L.J., Iturbide M.I., Torres Stone R.A., McGinley M., Raffaelli M., Carlo G. (2007). Acculturative stress, social support, and coping: Relations to psychological adjustment among Mexican American college students. Cultur. Divers. Ethnic Minor. Psychol..

[64] Xiao H., Zhang Y., Kong D., Li S., Yang N. (2020). The effects of social support on sleep quality of medical staff treating patients with coronavirus disease 2019 (COVID-19) in January and February 2020 in China. Med. Sci. Monit..

[65] Glozah F.N. (2015). Exploring Ghanaian adolescents’ meaning of health and wellbeing: A psychosocial perspective. Int. J. Qual. Stud. Health WellBeing.

[66] Huber R.S., Sifers S.K., Houlihan D., Youngblom R. (2012). Teacher support as a moderator of behavioral outcomes for youth exposed to stressful life events. Educ. Res. Int..

[67] Wright M.F., Wachs S. (2022). Self-isolation during the beginning of the COVID-19 pandemic and adolescents’ health outcomes: The moderating effect of perceived teacher support. Sch. Psychol..

[68] CBOS Raport z Badania pn. “Młodzież 2018”. Report from “Youth 2018”. https://www.cinn.gov.pl/portal?id=1475772.

[69] Zhou Q., Fan L., Yin Z. (2018). Association between family socioeconomic status and depressive symptoms among Chinese adolescents: Evidence from a National Household Survey. Psychiatry Res..

[70] Długosz P. (2022). Distance education and its impact on school performance: A cross-section study in Poland and Ukraine. Youth Cent. East. Eur..

[71] Schubert K.O., Clark S.R., Van L.K., Collinson J.L., Baune B.T. (2017). Depressive symptom trajectories in late adolescence and early adulthood: A systematic review. Aust. N. Z. J. Psychiatry.

[72] Magson N.R., Freeman J., Rapee R.M., Richardson C.E., Oar E.L., Fardouly J. (2021). Risk and Protective Factors for Prospective Changes in Adolescent Mental Health during the COVID-19 Pandemic. J. Youth Adolesc..

[73] Rose A.J., Rudolph K.D. (2006). A review of sex differences in peer relationship processes: Potential trade-offs for the emotional and behavioral development of girls and boys. Psychol. Bull..

[74] Sztompka P. (2000). Cultural Trauma: The Other Face of Social Change. Eur. J. Soc. Theory.

[75] Demertzis N., Eyerman R. (2020). COVID-19 as cultural trauma. Am. J. Cult. Sociol..

[76] Długosz P., Kryvachuk L., Shyyan O. (2021). Praktyki Społeczne w Czasie Pandemii COVID-19 wśród Polskiej i Ukraińskiej Młodzieży. Social Practices in the COVID-19 Pandemic among Polish and Ukrainian Youth.

[77] Wagener L.M., Furrow J.L., King P.E., Leffert N., Benson P. (2003). Religious involvement and developmental resources in youth. Rev. Relig. Res..

